# JAK3 and TYK2 Serve as Prognostic Biomarkers and Are Associated with Immune Infiltration in Stomach Adenocarcinoma

**DOI:** 10.1155/2020/7973568

**Published:** 2020-09-19

**Authors:** Lingkai Meng, Ling Ding, Yue Yu, Wang Li

**Affiliations:** ^1^NHC Key Laboratory of Hormones and Development, Tianjin Key Laboratory of Metabolic Diseases, Chu Hsien-I Memorial Hospital & Tianjin Institute of Endocrinology, Tianjin Medical University, Tianjin 300134, China; ^2^Department of Anesthesiology, Tianjin Union Medical Center, Tianjin 300191, China; ^3^University of Alberta, Edmonton, Alberta, Canada

## Abstract

**Background:**

Stomach adenocarcinoma (STAD) is one of the most common malignant tumors. The Janus kinases (JAKs) play a significant part in cellular biological process, inflammation, and immunity. The roles of JAKs in STAD are still not systematically described.

**Methods:**

A series of bioinformatics tools were used to clarify the role of JAKs in STAD.

**Results:**

JAK3/TYK2 levels were significantly increased in STAD during subgroup analyses based on gender, tumor grade, cancer stages, and nodal metastasis status. STAD patients with high levels of JAK3/TYK2 had poor overall survival, postprogression survival, and first progression. Immune infiltration revealed a significant correlation between JAK3/TYK2 expression and the abundance of immune cells as well as immune biomarker expression in STAD. JAK3/TYK2 was associated with the adaptive immune response, chemokine signaling pathway, and JAK-STAT signaling pathway.

**Conclusions:**

JAK3 and TYK2 serve as prognostic biomarkers and are associated with immune infiltration in STAD.

## 1. Introduction

Gastric cancer (GC) is one of the most common malignant tumors, with the fifth largest incidence and third largest mortality rate among all malignant tumors [1]. Stomach adenocarcinoma (STAD) is the most common subtype of GC, accounting for over 95% of all GC cases.

Although the identification of *Helicobacter pylori* has reduced the incidence of gastric cancer, it is estimated that 1,033,701 patients would be initially diagnosed with GC worldwide in 2018 [2]. Moreover, the molecular mechanisms concerning the tumorigenesis and progress of GC is far from clarified and the therapeutic measures for GC are limited, resulting in a poor patient prognosis. Furthermore, the overall survival of patients with advanced or metastatic GC is only approximately 1 year [3]. These sobering data illustrate a critical need for novel prognostic biomarkers and therapeutic targets for STAD.

Janus kinases (JAKs) are major activators of signal transducers and play a significant role in cellular biological processes, inflammation, and immunity [4–7]. JAK/STAT signaling is a key regulator of gene expression, transcriptional programs, and immune response. In all, four members have been identified in the JAK family: JAK1/2/3 and TYK2. Genetic alterations of JAKs are involved in tumor cell proliferation, migration, apoptosis, and metastasis in certain types of cancers [8]. Increasing evidence demonstrates JAKs as a prognostic biomarker and therapeutic target for many cancers or other diseases, such as JAK3 for renal cell carcinoma, JAK2 for acute lymphoblastic leukemia [9], JAK2 for skin cutaneous melanoma [10], and TYK2 for hepatocellular carcinoma [11]. However, specific functions of JAKs in STAD remain to be systematically described.

Therefore, we aimed to explore the expression of JAKs and prognostic value of the association between immune infiltration and JAKs in STAD. We further evaluated the correlation between JAK expression and the clinicopathological parameters of patients as well as immune infiltration in STAD. Our results may provide additional evidence about the prognostic biomarkers and therapeutic targets for STAD.

## 2. Materials and Methods

### 2.1. GEPIA

GEPIA is a novel bioinformatics web server for analyzing RNA sequencing expression data across The Cancer Genome Atlas Program (TCGA) cancers [12]. TCGA is a landmark cancer genomics program that has molecularly characterized more than 20,000 primary cancers and matched normal samples spanning 33 cancer types. Tumor/normal differential expression analysis of JAKs in STAD was explored using the TCGA STAD dataset (*n* = 415) in GEPIA with analysis of variance (ANOVA). A *P* value less than 0.05 indicated statistical significance.

### 2.2. UALCAN

UALCAN is designed for gene expression analysis, prognosis analysis, and methylation analysis based on the data from TCGA and Clinical Proteomic Tumor Analysis Consortium (CPTAC) [13]. In the current study, the correlation between JAK3 and TYK2 expression and the clinicopathological parameters of STAD patients, including the race, gender, age, *H*. *pylori* infection status, histological subtype, tumor grade, cancer stage, and nodal metastasis status of patients, were analyzed using the TCGA STAD dataset (*n* = 415). A *P* value less than 0.05 indicated statistical significance.

### 2.3. The Kaplan–Meier Plotter (KM Plotter)

The KM plotter is designed for the prognostic analysis of 54 k genes (mRNA, miRNA, and protein) in certain types of cancers including breast, lung, and gastric cancer [14]. Here, the significance of JAK3 and TYK2 in determining the overall survival (OS), postprogression survival (PPS), and first progression (FP) of STAD was analyzed using the Kaplan–Meier curve. The medium value of the JAK3 and TYK2 expressions was used to split patients into high-/low-expression groups.

### 2.4. cBioPortal

cBioPortal is a cancer genomics portal designed for exploring multidimensional cancer genomics data using the TCGA dataset [15]. We used cBioPortal to explore, visualize, and analyze the genetic alterations and mutations of JAK3 and TYK2 in STAD using the TCGA STAD dataset (*n* = 415). Furthermore, mRNA expression *z* scores (RNA Seq V2 RSEM) were obtained (*z* score threshold, ±2.0). Protein expression *z* scores (RPPA) were also obtained (*z* score threshold, ±2.0).

### 2.5. LinkedOmics

LinkedOmics is a bioinformatics web portal designed for accessing, analyzing, and comparing cancer multiomics data of various cancer types [16]. Complete data of 415 TCGA STAD patients were used to explore JAK3- and TYK2-associated genes via the Spearman correlation analysis. Moreover, Gene Set Enrichment Analysis (GSEA) was performed to explore JAK3- and TYK2-associated functions (GO analysis and KEGG pathway analysis) in STAD, with the minimum number of genes being three and the *P* value threshold being 0.05. The transcription factor targets of JAK3 and TYK2 were also analyzed via GSEA.

### 2.6. TIMER

TIMER is a comprehensive resource for the systematic analysis of immune infiltrates across diverse cancer types [17]. In the current study, the Spearman correlation analysis was used to explore the correlation between the expression levels of JAK3/TYK2 and the abundance of immune cell infiltrates and the expression of gene biomarkers of immune cells [18–20]. The two-sided Wilcoxon rank-sum test was used to evaluate the effect of somatic copy number alterations (SCNAs) of JAK3/TYK2 on immune cell infiltrates. A *P* value less than 0.05 indicated statistical significance.

## 3. Results

### 3.1. JAK Expression in STAD

**Figure 1:**
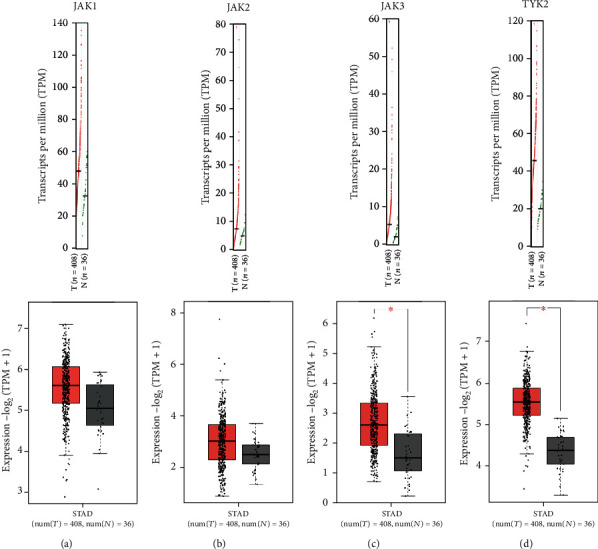
The expression of JAKs in STAD (GEPIA). The expression of JAK3 and TYK2 were significantly elevated in STAD tissues at mRNA level. STAD: stomach adenocarcinoma; ^∗^*P* < 0.05; T: tumor tissues; N: normal tissues.

**Figure 2:**
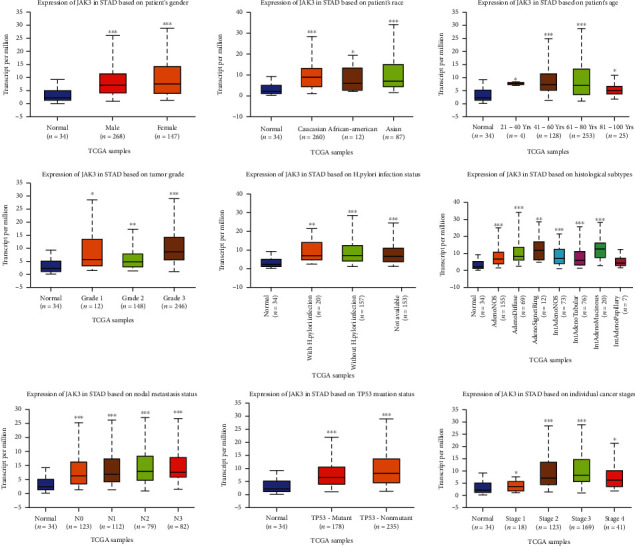
The expression of JAK3 in STAD in subgroup analyses (UALCAN). Subgroup analyses were performed based on patients' race, patients' gender, patients' age, H. pylori infection status, histological subtypes, tumor grade, individual cancer stages, and nodal metastasis status. STAD: stomach adenocarcinoma; ^∗^*P* < 0.05, ^∗∗^*P* < 0.01, and ^∗∗∗^*P* < 0.001.

**Figure 3:**
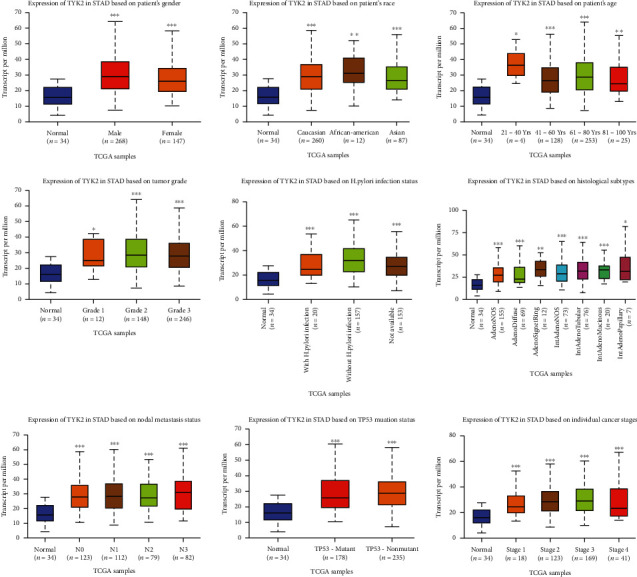
The expression of TYK2 in STAD in subgroup analyses (UALCAN). Subgroup analyses were performed based on patients' race, patients' gender, patients' age, H. pylori infection status, histological subtypes, tumor grade, individual cancer stages, and nodal metastasis status. STAD: stomach adenocarcinoma; ^∗^*P* < 0.05, ^∗∗^*P* < 0.01, and ^∗∗∗^*P* < 0.001.

The level of JAKs in primary STAD was first determined using GEPIA. As shown in Figure 1, the expression levels of JAK3 (Figure 1(c), *P* < 0.05) and TYK2 (Figure 1(d), *P* < 0.05) were significantly elevated in STAD tissues compared with normal tissues. However, there was no difference in the expression levels of JAK1 (Figure 1(a)**)** and JAK2 (Figure 1(b)) between STAD tissues and normal tissues. We then analyzed the correlation between the expression levels of JAK3/TYK2 and the clinicopathological parameters of STAD patients. As expected, the mRNA levels of JAK3 were significantly increased in STAD during subgroup analyses based on the race, gender, age, *H*. *pylori* infection status, histological subtype, tumor grade, cancer stage, and nodal metastasis status of patients (Figure 2). The same results were obtained for TYK2, and the mRNA levels of JAK3 were significantly increased in STAD during subgroup analyses based on the race, gender, age, *H*. *pylori* infection status, histological subtype, tumor grade, individual cancer stage, and nodal metastasis status of patients (Figure 3). Therefore, JAK3 and TYK2 may play a significant role in the tumorigenesis, progression, and aggressiveness of STAD.

### 3.2. JAK3/TYK2 As a Prognostic Biomarker in STAD

**Figure 4:**
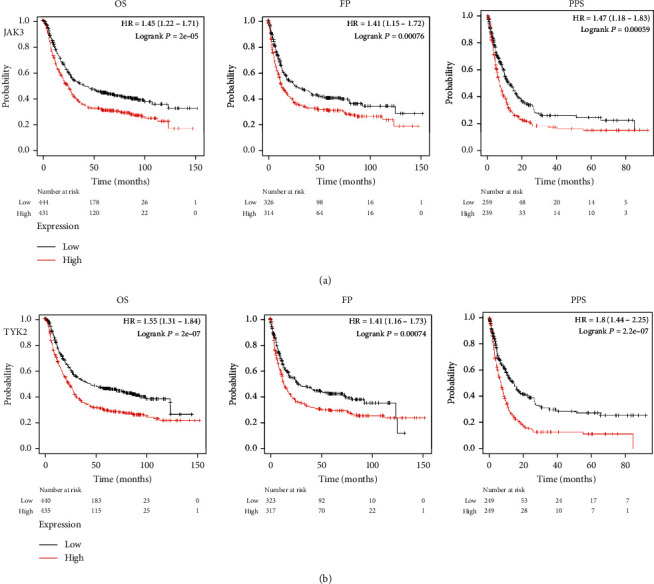
The prognostic value of JAK3/TYK2 in STAD (KM plotter). (a) STAD patients with high mRNA level of JAK3 had worse OS, PF, and PPS. (b) STAD patients with high mRNA level of TYK2 had worse OS, PF, and PPS. All the analyses were performed with Kaplan–Meier analysis. HR: hazard ratio; OS: overall survival; PPS: postprogression survival; FP: first progression.

The prognostic value of JAK3/TYK2 in STAD was evaluated using the KM plotter. We found that STAD patients with high JAK3 levels had poor OS (HR = 1.45 (1.22-1.71), *P* = 2*e*^−5^), FP (HR = 1.41 (1.15-1.72), *P* = 0.00076), and PPS (HR = 1.47 (1.18-1.83), *P* = 0.00059) (Figure 4(a)). Moreover, even STAD patients with high TYK2 levels had poor OS (HR = 1.55 (1.31-1.84), *P* = 4*e*^−7^), FP (HR = 1.41 (1.16-1.73), *P* = 0.00074), and PPS (HR = 1.8 (1.44-2.25), *P* = 2.2*e*^−7^). Thus, JAK3/TYK2 served as a prognostic biomarker in STAD (Figure 4(b)).

To better understand how the expression levels of JAK3 and TYK2 impact the prognosis of STAD patients, we also analyzed the correlation between the expression of JAK3 and TYK2 and clinical characteristics of TCGA STAD patients using the KM plotter. JAK3 and TYK2 overexpression was associated with worse OS (Table 1) and PFS (Table 2) in male and female patients as well as in patients with intestinal and diffuse type Lauren classification (*P* < 0.05). Further, the overexpression of JAK3 and TYK2 was associated with worse FP (Table 3) in male and female patients (*P* < 0.05). STAD patients with poor differentiation and high JAK3 levels had worse OS (Table 1) and PFS (Table 2), although the *P* value in OS analysis was 0.059. We further found that the overexpression of JAK3 and TYK2 was associated with worse OS (Table 1) and PFS (Table 2) in patients with stage 2 and 3 disease (*P* < 0.05). STAD patients with regional lymph node metastasis (N stage 1 or 1+2+3) and high JAK3 expression had significantly worse OS (Table 1), PFS (Table 2), and PF (Table 3). Similarly, STAD patients with regional lymph node metastasis (N stage 1, 2, or 1+2+3) and high TYK2 expression levels had worse OS Table 1), PFS (Table 2), and PF (Table 3). Therefore, JAK3/TYK2 level can impact the prognosis of STAD patients with lymph node metastasis.

### 3.3. Genetic Alterations of JAK3/TYK2 in STAD

**Figure 5:**
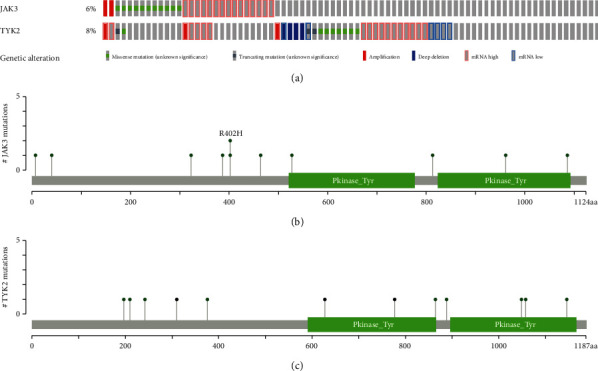
Genetic alteration of JAK3/TYK2 in STAD (cBioPortal). (a) OncoPrint of JAK3/TYK2 alterations in STAD. (b, c) Mutation sites of JAK3/TYK2 in STAD.

cBioPortal was used to determine the genetic alterations of JAK3/TYK2 in STAD. We found that JAK3 and TYK2 were altered in 6% and 8% of all TCGA STAD cases, respectively (Figure 5(a)).

Genetic alterations of JAK3 and TYK2 in STAD comprised missense mutation, truncating mutation, amplification, deep deletion, high mRNA levels, and low mRNA levels. Thus, mutation is the most common type of JAK3/TYK2 genetic alteration. The mutation sites of JAK3/TYK2 in STAD are shown in Figures 5(b) and 5(c).

### 3.4. JAK3/TYK2 Correlated with Immune Infiltration in STAD

**Figure 6:**
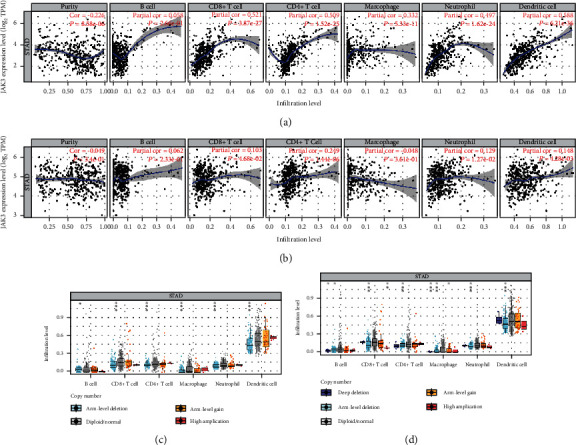
The correlation between JAK3/TYK2 and immune infiltration (TIMER). (Aa, b) The correlation between JAK3/TYK2 expression and the abundance of CD8+ T cells, CD4+ T cells, macrophage, neutrophils, and dendritic cells. (c, d) The correlation between SCNA of JAK3/TYK2 and immune cell infiltration. SCNA: somatic copy number alterations; ^∗^*P* < 0.05, ^∗∗^*P* < 0.01, and ^∗∗∗^*P* < 0.001.

An increasing number of studies have suggested an interaction between immune response and pathophysiological processes [21, 22]. Moreover, JAKs play a critical role in immune regulation by invoking intracellular signaling pathways in cancers [23]. Therefore, we next evaluated the correlation between JAK3/TYK2 and immune infiltration in STAD. As shown in Figure 6, JAK3 levels showed a positive correlation with the abundance of CD8+ T cells (Cor = 0.521, *P* = 3.87*e*^−27^), CD4+ T cells (Cor = 0.509, *P* = 1.52*e*^−25^), macrophages (Cor = 0.332, *P* = 5.33*e*^−12^), neutrophils (Cor = 0.497, *P* = 1.62*e*^−24^), and dendritic cells (Cor = 0.588, *P* = 6.21*e*^−36^) (Figure 6(a)). We also found a positive correlation between TYK2 levels and the abundance of CD8+ T cells (Cor = 0.103, *P* = 0.0468), CD4+ T cells (Cor = 0.249, *P* = 1.44*e*^−06^), neutrophils (Cor = 0.129, *P* = 0.0127), and dendritic cells (*Cor* = 0.148, *P* = 0.00428) (Figure 6(b)). Interestingly, SCNA of JAK3/TYK2 could partially inhibit immune infiltration in STAD (Figures 6(c) and 6(d)).

We also evaluated the correlation between JAK3/TYK2 and immune biomarkers in STAD. Previous studies have reported these biomarkers of immune cells [18–20]. As expected, the expression levels of JAK3/TYK2 were positively correlated with the expression levels of immune biomarkers in STAD (Tables 4 and 5). We found that the expression levels of biomarkers of CD8+ T cells (CD8A and CD8B), T cells (CD3D, CD3E, and CD2), B cells (CD19 and CD79A), monocytes (CD86 and CD115), and TAMs (CD68 and IL10) positively correlated with the expression levels of JAK3 and TYK2 in STAD. The expression levels of INOS, IRF5, CD163, VSIG4, MS4A4A, CD11b, and CCR7 were positively correlated with JAK3/TYK2 levels in STAD. All biomarkers of natural killer cells (KIR2DL1, KIR2DL3, KIR2DL4, KIR3DL1, KIR3DL2, KIR3DL3, and KIR2DS4) showed positive correlation with JAK3 expression. Similarly, all biomarkers of dendritic cells (KIR2DL1, KIR2DL3, KIR2DL4, KIR3DL1, KIR3DL2, KIR3DL3, and KIR2DS4), Th1 cells (TBX21, STAT4, STAT1, IIFNG, and TNF), Th2 cells (GATA3, STAT6, STAT5A, and IL13), and Tfh cells (BCL6 and IL21) showed a positive correlation with the JAK3 and TYK2 expressions. Moreover, levels of immune biomarkers of Treg cells (FOXP3, CCR8, and STAT5B) and T cell exhaustion (PD-1, CTLA4, LAG3, TIM-3, and GZMB) were positively associated with JAK3 and TYK2 levels. These results indicate that JAK3 and TYK2 played a vital role in immune escape in the STAD microenvironment.

### 3.5. Enrichment Analysis of JAK3/TYK2 in STAD

The function module of LinkedOmics was used to performed enrichment analysis of JAK3/TYK2 in STAD. In all, 7855 genes (dark red dots) were positively correlated with JAK3, whereas 4687 genes (dark green dots) were negatively correlated with JAK3 in STAD (Supplementary Figure 2A, *P* < 0.05). Further, 50 significant gene sets that positively and negatively correlated with JAK3 in STAD are presented in Supplementary Figure 2A-2C, respectively. Enrichment analysis performed via GSEA suggested that JAK3 is associated with adaptive immune response, protein transmembrane transport, DNA damage response, DNA damage detection, preribosomal structure, respiratory chain, cytokine binding, translation factor activity, RNA binding, snoRNA binding, and tRNA binding during GO analysis (Supplementary Figure 2D-2F). Moreover, KEGG analysis revealed that JAK3 was involved in cytokine-cytokine receptor interactions, chemokine signaling pathway, NF-kappa B signaling pathway, Th17 cell differentiation, and T cell receptor signaling pathway and that JAK3 was associated with cell adhesion molecules (CAMs) (Supplementary Figure 2G and Supplementary Figure 3).

The results of enrichment analysis of TYK2 in STAD are shown Supplementary Figure 4. We found that 5756 genes (dark red dots) were positively correlated with TYK2, whereas 3993 genes (dark green dots) were negatively correlated with TYK2 in STAD (Supplementary Figure 4A, *P* < 0.05). Further, 50 significant gene sets that positively and negatively correlated with TYK2 in STAD are presented in Supplementary Figure 4B and 4C, respectively. Enrichment analysis performed by GSEA suggested that TYK2 was associated with the regulation of leukocyte activation, adaptive immune responses, translational initiation, mitochondrial matrix, ribosomal structure, translation factor activity, cytokine receptor activity, rRNA binding, and protein transporter activity during GO analysis (Supplementary Figure 4D-4F). Furthermore, KEGG analysis revealed that JAK3 was associated with ribosomal structure, cytokine-cytokine receptor interaction, JAK-STAT signaling pathway, RNA transport, CAMs, and Th1 and Th2 cell differentiation (Supplementary Figure 4G and Supplementary Figure 5).

## 4. Discussion

Increasing evidence has revealed that JAKs play an important role in the regulation of cytokine signaling, thus affecting basic cellular mechanisms, such as cell invasion, proliferation, apoptosis, and cellular immunity [5, 24]. Moreover, JAK-associated signaling pathways are associated with tumorigenesis and progression of cancers, including lung cancer, renal cell carcinoma, and lung cancer [25–27]. However, specific functions of the JAK family in STAD remain to be systematically described. Therefore, our study was conducted to clarify the role of JAKs in STAD.

In this study, we found that the expression levels of JAK3 and TYK2 were higher in tumor tissues than in normal tissues in STAD. Further analysis revealed that JAK3 and TYK2 served as prognostic biomarkers in STAD and were associated with tumorigenesis, progression, and metastasis of STAD. Previous studies have also suggested that JAKs serve as biomarkers in certain types of cancers. In clear cell renal cell carcinoma, JAK3 acted as a novel biomarker and was associated with immune infiltration [26]. Another study revealed that JAK2 was a prognostic biomarker in skin cutaneous melanoma and was involved in gene regulation [10]. Moreover, JAK2 and TYK2 were suggested to be potential biomarkers for the diagnosis of hepatocellular carcinoma.

Another significant finding of our study is that JAK3 and TYK2 were associated with the abundance of immune cells, including CD8+ T cells, CD4+ T cells, neutrophils, and dendritic cells. Moreover, the expression levels of JAK3/TYK2 were positively correlated with the expression levels of immune biomarkers in STAD, demonstrating that JAK3 and TYK2 may play a vital role in immune escape in the STAD microenvironment. Previous studies have also clarified the significant role of JAK3 and TYK2 in the tumor microenvironment and immune response. JAK3 has been reported to be involved in hematopoiesis during T cell development by mediating innate and adaptive immunity-associated signaling [28]. Another study has reported that JAK3 deficiency can inhibit the development of innate lymphoid cells [29]. In lung cancer, JAK3 variants can promote PD-L1 induction in the tumor immune microenvironment and JAK3 activation may contribute to the long-term efficacy of PD-L1 [30]. A CTLA-4-TYK2-STAT3 axis has been reported in B cell lymphoma cells and tumor-associated B cells and is relevant to immune checkpoint therapy [31].

In this study, enrichment analysis was performed, which revealed the functions and pathways of JAK3 and TYK2 in STAD, indicating that JAK3 and TYK2 were mainly associated with adaptive immune responses, translational initiations, DNA damage responses, chemokine signaling pathway, NF-kappa B signaling pathway, ribosomal structure, and JAK-STAT signaling pathway. It is well known that NF-kappa B signaling pathway is involved in inflammation and innate immunity and plays a vital role in cancer initiation and progression [32]. Moreover, NF-*κ*B suppression can inhibit tumor cell growth and promote cell apoptosis in cholangiocarcinoma [33]. Increasing evidence has also highlighted the significant role of JAK/STAT/NF-*κ*B signaling pathway in the immune response, axial spondyloarthritis, type 2 diabetes, metabolic disorders, and cancers [34–38]. Thus, JAK3 and TYK2 may exert functions in STAD via JAK-STAT and NF-*κ*B signaling pathway.

This study has some limitations. First, in our study, we performed analysis at an mRNA level; it would be better to verify our results at a protein level. Furthermore, validation of our results by performing *in vivo* and *in vitro* experiments is warranted.

In conclusion, our results demonstrated that JAK3 and TYK2 serve as prognostic biomarkers and are associated with immune infiltration in STAD, providing additional data about biomarkers, STAD prognosis, and therapy.

## Figures and Tables

**Table 1:** Correlation of JAK3/TYK2 mRNA expression and overall survival in STAD with different clinicopathological factors (Kaplan–Meier plotter).

Pathological parameters	Overall survival					
	JAK3			TYK2		
	*N*	Hazard radio	*P* value	*N*	Hazard radio	*P* value
Sex						
Female	236	1.93 (1.36-2.74)	0.00017	236	1.5 (1.06-2.14)	0.023
Male	544	1.59 (1.23-2.04)	0.00028	544	1.86 (1.5-2.3)	1*e*^−8^
Stage						
1	67	3.61 (1.35-9.65)	0.0062	67	2.02 (0.75-5.44)	0.16
2	140	2.39 (1.3-4.38)	0.0037	140	1.92 (1.03-3.56)	0.036
3	305	1.56 (0.17-2.09)	0.0023	305	1.66 (1.22-2.26)	0.001
4	148	0.76 (0.49-1.17)	0.21	148	0.71 (0.48-1.06)	0.091
Stage T						
2	241	1.47 (0.93-2.3)	0.094	241	1.28 (0.82-2)	0.27
3	204	0.78 (0.54-1.13)	0.19	204	1.47 (0.97-2.22)	0.065
4	38	0.33 (0.1-1.11)	0.059	38	0.67 (0.28-1.59)	0.36
Stage N						
0	74	2.03 (0.6-6.85)	0.24	74	1.75 (0.75-4.07)	0.19
1	225	2.63 (1.49-4.66)	0.00054	225	1.63 (1.07-2.48)	0.02
2	121	0.72 (0.45-1.15)	0.17	121	0.64 (0.41-1)	0.048
3	76	0.7 (0.37-1.31)	0.26	76	0.64 (0.38-1.1)	0.11
1+2+3	422	1.35 (1.01-1.8)	0.043	422	1.38 (1.3-1.54)	0.03
Stage M						
0	444	1.3 (0.97-1.75)	0.082	444	1.32 (1-1.76)	0.053
1	56	0.77 (0.4-1.5)	0.44	56	0.37 (0.16-0.82)	0.011
Lauren classification						
Intestinal	320	3.32 (1.5-3.59)	0.0001	320	1.74 (1.27-2.39)	0.00047
Diffuse	241	1.38 (0.97-1.97)	0.07	241	1.15 (0.81-1.62)	0.44
Differentiation						
Poor	165	1.5 (0.98-2.3)	0.059	165	0.83 (0.55-1.23)	0.34
Moderate	67	0.57 (0.29-1.11)	0.096	67	0.56 (0.29-1.07)	0.075

**Table 2:** Correlation of JAK3/TYK2 mRNA expression and postprogression survival in STAD with different clinicopathological factors (Kaplan–Meier plotter).

Pathological parameters	Post progression survival					
	JAK3			TYK2		
	*N*	Hazard radio	*P* value	*N*	Hazard radio	*P* value
Sex						
Female	149	1.84 (1.19-2.85)	0.0053	149	2.36 (1.49-3.73)	0.00015
Male	348	1.71 (1.31-2.22)	0.000055	348	2.32 (1.79-3.02)	1.1*e*^−10^
Stage						
1	31	2.86 (0.5-16.46)	0.22	31	1.66 (0.37-7.43)	0.51
2	105	2.5 (1.28-4.87)	0.0053	105	2.39 (1.23-4.64)	0.0081
3	142	1.45 (0.92-2.28)	0.1	142	2.47 (1.6-3.83)	2.7*e*^−5^
4	104	1.66 (1.01-2.72)	0.045	104	0.68 (0.42-1.11)	0.12
Stage T						
2	196	1.66 (1.05-2.64)	0.029	196	1.72 (1.09-2.73)	0.019
3	150	1.21 (0.8-1.82)	0.36	150	1.91 (1.17-3.12)	0.0083
4	29	0.45 (0.15-1.38)	0.15	29	0.61 (0.22-1.64)	0.32
Stage N						
0	41	2.59 (0.72-9.35)	0.13	41	2.57 (0.77-8.57)	0.11
1	169	2.52 (1.6-3.98)	0.000039	169	2.48 (1.58-3.91)	4.8*e*^−5^
2	105	0.7 (0.43-1.15)	0.16	105	1.67 (0.99-2.81)	0.054
3	63	1.65 (0.86-3.18)	0.13	63	0.53 (0.29-0.95)	0.032
1+2+3	337	1.44 (1.06-1.95)	0.02	337	1.62 (1.21-2.17)	0.0011
Stage M						
0	342	1.38 (1-1.9)	0.051	342	1.91 (1.4-2.6)	2.7*e*^−5^
1	36	2.35 (1.08-5.13)	0.028	36	0.73 (0.33-1.63)	0.44
Lauren classification						
Intestinal	192	1.69 (1.08-2.66)	0.02	192	1.86 (1.22-2.84)	0.0037
Diffuse	176	1.5 (0.99-2.26)	0.053	176	1.55 (1.05-2.29)	0.028
Differentiation						
Poor	49	3.3 (1.59-6.88)	0.00076	49	1.68 (0.82-3.41)	0.15
Moderate	24	2.06 (0.75-5.63)	0.15	24	0.63 (0.26-1.56)	0.32

**Table 3:** Correlation of JAK3/TYK2 mRNA expression and first progression in STAD with different clinicopathological factors (Kaplan–Meier plotter).

Pathological parameters	First progression					
	JAK3			TYK2		
	*N*	Hazard radio	*P* value	*N*	Hazard radio	*P* value
Sex						
Female	201	2.02 (1.38-2.95)	0.00021	201	1.37 (0.94-2)	0.097
Male	437	1.42 (1.1-1.84)	0.0076	437	2.07 (1.6-2.68)	1.4*e*^−8^
Stage						
1	60	2.37 (0.79-7.1)	0.11	60	0.54 (0.18-1.68)	0.28
2	131	1.5 (0.79-2.84)	0.21	131	1.42 (0.77-2.61)	0.25
3	186	1.52 (1.04-2.24)	0.031	186	1.36 (0.94-1.97)	0.1
4	141	0.64 (0.41-1.02)	0.057	141	0.73 (0.48-1.11)	0.14
Stage T						
2	239	1.51 (0.92-2.45)	0.097	239	1.21 (0.8-1.83)	0.36
3	204	0.7 (0.49-1.01)	0.054	204	1.35 (0.9-2.02)	0.15
4	39	0.46 (0.2-1.09)	0.072	39	0.77 (0.36-1.66)	0.5
Stage N						
0	72	2.22(0.66-7.49)	0.19	72	1.56 (0.66-3.65)	0.3
1	222	2.27 (1.34-3.82)	0.0016	222	1.45 (0.98-2.15)	0.059
2	125	0.84 (0.53-1.32)	0.44	125	0.59 (0.38-0.91)	0.015
3	76	1.31 (0.72-2.37)	0.37	76	0.73 (0.41-1.33)	0.3
1+2+3	423	1.32 (1-1.74)	0.049	423	0.89 (0.67-1.17)	0.4
Stage M						
0	443	1.35 (0.98-1.85)	0.066	443	1.21 (0.92-1.59)	0.17
1	56	0.6 (0.33-1.11)	0.099	56	0.41 (0.19-0.92)	0.026
Lauren classification						
Intestinal	263	1.74 (1.15-2.62)	0.0078	263	1.28 (0.88-1.88)	0.2
Diffuse	231	1.25(0.87-1.79)	0.22	231	0.85 (0.59-1.24)	0.4
Differentiation						
Poor	121	1.32 (0.84-2.09)	0.23	121	0.67 (0.41-1.08)	0.095
Moderate	67	0.63 (0.33-1.19)	0.15	67	0.6 (0.32-1.13)	0.11

**Table 4:** Correlation analysis between JAK3 and gene biomarkers of immune cells in STAD (TIMER).

Description	Biomarkers	STAD			
		None		Purity	
		Cor	*P* value	Cor	*P* value
CD8+ T cell	CD8A	0.7	^∗∗∗^	0.684	^∗∗∗^
	CD8B	0.553	^∗∗∗^	0.539	^∗∗∗^

T cell (general)	CD3D	0.711	^∗∗∗^	0.696	^∗∗∗^
	CD3E	0.735	^∗∗∗^	0.73	^∗∗∗^
	CD2	0.701	^∗∗∗^	0.685	^∗∗∗^

B cell	CD19	0.658	^∗∗∗^	0.648	^∗∗∗^
	CD79A	0.629	^∗∗∗^	0.605	^∗∗∗^

Monocyte	CD86	0.562	^∗∗∗^	0.536	^∗∗∗^
	CD115(CSF1R)	0.541	^∗∗∗^	0.527	^∗∗∗^

TAM	CCL2	0.441	^∗∗∗^	0.404	^∗∗∗^
	CD68	0.318	^∗∗∗^	0.294	^∗∗∗^
	IL10	0.482	^∗∗∗^	0.45	^∗∗∗^

M1 macrophage	INOS (NOS2)	0.135	^∗∗^	0.129	^∗^
	IRF5	0.401	^∗∗∗^	0.378	^∗∗∗^
	COX2(PTGS2)	0.036	0.465	0.006	0.915

M2 macrophage	CD163	0.482	^∗∗∗^	0.466	^∗∗∗^
	VSIG4	0.389	^∗∗∗^	0.382	^∗∗∗^
	MS4A4A	0.474	^∗∗∗^	0.451	^∗∗∗^

Neutrophils	CD66b (CEACAM8)	0.054	0.269	0.053	0.307
	CD11b (ITGAM)	0.563	^∗∗∗^	0.554	^∗∗∗^
	CCR7	0.725	^∗∗∗^	0.708	^∗∗∗^

Natural killer cell	KIR2DL1	0.283	^∗∗^	0.261	^∗∗∗^
	KIR2DL3	0.264	^∗∗∗^	0.217	^∗∗∗^
	KIR2DL4	0.301	^∗∗∗^	0.268	^∗∗∗^
	KIR3DL1	0.29	^∗∗∗^	0.268	^∗∗∗^
	KIR3DL2	0.433	^∗∗∗^	0.396	^∗∗∗^
	KIR3DL3	0.103	^∗^	0.103	^∗^
	KIR2DS4	0.283	^∗∗^	0.253	^∗∗∗^

Dendritic cell	HLA-DPB1	0.581	^∗∗∗^	0.556	^∗∗∗^
	HLA-DQB1	0.466	^∗∗∗^	0.418	^∗∗∗^
	HLA-DRA	0.496	^∗∗∗^	0.471	^∗∗∗^
	HLA-DPA1	0.495	^∗∗∗^	0.464	^∗∗∗^
	BDCA-1(CD1C)	0.52	^∗∗∗^	0.482	^∗∗∗^
	BDCA-4(NRP1)	0.472	^∗∗∗^	0.454	^∗∗∗^
	CD11c (ITGAX)	0.64	^∗∗∗^	0.625	^∗∗∗^

Th1	T-bet (TBX21)	0.753	^∗∗∗^	0.753	^∗∗∗^
	STAT4	0.759	^∗∗∗^	0.757	^∗∗∗^
	STAT1	0.466	^∗∗∗^	0.487	^∗∗∗^
	IFN-g (IFNG)	0.438	^∗∗∗^	0.429	^∗∗∗^
	TNF-a (TNF)	0.361	^∗∗∗^	0.322	^∗∗∗^

Th2	GATA3	0.633	^∗∗∗^	0.625	^∗∗∗^
	STAT6	0.296	^∗∗∗^	0.321	^∗∗∗^
	STAT5A	0.581	^∗∗∗^	0.576	^∗∗∗^
	IL13	0.214	^∗∗∗^	0.218	^∗∗∗^

Tfh	BCL6	0.426	^∗∗∗^	0.413	^∗∗∗^
	IL21	0.384	^∗∗∗^	0.362	^∗∗∗^

Th17	STAT3	0.455	^∗∗∗^	0.467	^∗∗∗^
	IL17A	0.075	0.12	0.087	0.0919

Treg	FOXP3	0.711	^∗∗∗^	0.679	^∗∗∗^
	CCR8	0.657	^∗∗∗^	0.644	^∗∗∗^
	STAT5B	0.534	^∗∗∗^	0.545	^∗∗∗^
	TGFb (TGFB1)	0.536	^∗∗∗^	0.52	^∗∗∗^

T cell exhaustion	PD-1 (PDCD1)	0.725	^∗∗∗^	0.718	^∗∗∗^
	CTLA4	0.645	^∗∗∗^	0.623	^∗∗∗^
	LAG3	0.608	^∗∗∗^	0.597	^∗∗∗^
	TIM-3 (HAVCR2)	0.567	^∗∗∗^	0.545	^∗∗∗^
	GZMB	0.449	^∗∗∗^	0.407	^∗∗∗^

^∗^ *P* < 0.05, ^∗∗^*P* < 0.001, and ^∗∗∗^*P* < 0.001.

**Table 5:** Correlation analysis between TYK2 and gene biomarkers of immune cells in STAD (TIMER).

Description	Biomarkers	STAD			
		None		Purity	
		Cor	*P* value	Cor	*P* value
CD8+ T cell	CD8A	0.298	^∗∗∗^	0.318	^∗∗∗^
	CD8B	0.161	^∗∗^	0.166	^∗∗^

T cell (general)	CD3D	0.214	^∗∗∗^	0.233	^∗∗∗^
	CD3E	0.295	^∗∗∗^	0.32	^∗∗∗^
	CD2	0.257	^∗∗∗^	0.278	^∗∗∗^

B cell	CD19	0.296	^∗∗∗^	0.309	^∗∗∗^
	CD79A	0.231	^∗∗∗^	0.242	^∗∗∗^

Monocyte	CD86	0.213	^∗∗∗^	0.219	^∗∗∗^
	CD115(CSF1R)	0.302	^∗∗∗^	0.296	^∗∗∗^

TAM	CCL2	0.039	0.432	0.028	0.586
	CD68	0.267	^∗∗∗^	0.265	^∗∗∗^
	IL10	0.288	^∗∗∗^	0.286	^∗∗∗^

M1 macrophage	INOS (NOS2)	0.17	^∗∗∗^	0.165	^∗∗^
	IRF5	0.37	^∗∗∗^	0.363	^∗∗∗^
	COX2(PTGS2)	0.009	0.858	0	1

M2 macrophage	CD163	0.31	^∗∗∗^	0.304	^∗∗∗^
	VSIG4	0.159	^∗∗^	0.152	^∗∗^
	MS4A4A	0.18	^∗∗∗^	0.174	^∗∗∗^

Neutrophils	CD66b (CEACAM8)	0.018	0.713	0.028	0.591
	CD11b (ITGAM)	0.411	^∗∗∗^	0.413	^∗∗∗^
	CCR7	0.31	^∗∗∗^	0.331	^∗∗∗^

Natural killer cell	KIR2DL1	0.036	0.463	0.055	0.283
	KIR2DL3	0.037	0.448	0.046	0.372
	KIR2DL4	0.158	^∗∗^	0.178	^∗∗∗^
	KIR3DL1	0.114	0.02	0.128	0.0126
	KIR3DL2	0.145	^∗∗^	0.151	^∗∗^
	KIR3DL3	0.094	0.0554	0.114	^∗^
	KIR2DS4	0.09	0.0673	0.104	^∗^

Dendritic cell	HLA-DPB1	0.26	^∗∗∗^	0.274	^∗∗∗^
	HLA-DQB1	0.249	^∗∗∗^	0.271	^∗∗∗^
	HLA-DRA	0.263	^∗∗∗^	0.278	^∗∗∗^
	HLA-DPA1	0.259	^∗∗∗^	0.269	^∗∗∗^
	BDCA-1(CD1C)	0.175	^∗∗∗^	0.159	^∗∗^
	BDCA-4(NRP1)	0.26	^∗∗∗^	0.244	^∗∗∗^
	CD11c (ITGAX)	0.393	^∗∗∗^	0.404	^∗∗∗^

Th1	T-bet (TBX21)	0.372	^∗∗∗^	0.397	^∗∗∗^
	STAT4	0.31	^∗∗∗^	0.33	^∗∗∗^
	STAT1	0.373	^∗∗∗^	0.393	^∗∗∗^
	IFN-g (IFNG)	0.24	^∗∗∗^	0.265	^∗∗∗^
	TNF-a (TNF)	0.254	^∗∗∗^	0.255	^∗∗∗^

Th2	GATA3	0.218	^∗∗∗^	0.235	^∗∗∗^
	STAT6	0.435	^∗∗∗^	0.438	^∗∗∗^
	STAT5A	0.546	^∗∗∗^	0.561	^∗∗∗^
	IL13	0.101	0.039	0.118	0.0213

Tfh	BCL6	0.232	^∗∗∗^	0.233	^∗∗∗^
	IL21	0.216	^∗∗∗^	0.211	^∗∗∗^

Th17	STAT3	0.457	^∗∗∗^	0.456	^∗∗∗^
	IL17A	0.057	0.25	0.068	0.189

Treg	FOXP3	0.439	^∗∗∗^	0.467	^∗∗∗^
	CCR8	0.372	^∗∗∗^	0.379	^∗∗∗^
	STAT5B	0.493	^∗∗∗^	0.489	^∗∗∗^
	TGFb (TGFB1)	0.288	^∗∗∗^	0.293	^∗∗∗^

T cell exhaustion	PD-1 (PDCD1)	0.411	^∗∗∗^	0.448	^∗∗∗^
	CTLA4	0.322	^∗∗∗^	0.347	^∗∗∗^
	LAG3	0.27	^∗∗∗^	0.296	^∗∗∗^
	TIM-3 (HAVCR2)	0.306	^∗∗∗^	0.314	^∗∗∗^
	GZMB	0.159	^∗∗^	0.174	^∗∗∗^

^∗^ *P* < 0.05, ^∗∗^*P* < 0.001, and ^∗∗∗^*P* < 0.001.

## Data Availability

The analyzed data sets generated during the study are available from the corresponding author on reasonable request.

## Supplementary Materials

Supplementary Materials Supplementary Figure 1 The expression of JAKs in STAD (UALCAN). The expressions of JAK1, JAK2, JAK3, and TYK2 were significantly elevated in STAD tissues at mRNA level. STAD: stomach adenocarcinoma; ^∗∗∗^*P* < 0.001. Supplementary Figure 2. The enrichment analysis of JAK3 in STAD (LinkedOmics). (A) A Pearson test was used to analyze correlations between JAK3 and genes differentially expressed in STAD. (B, C) Heat maps showing genes positively and negatively correlated with JAK3 in STAD (Top 50). Red indicates positively correlated genes, and green indicates negatively correlated genes. (D–F) Heat map of GO enrichment in CC terms, BP terms, and MF terms. (G) KEGG pathways analysis. GO and KEGG were performed by Gene Set Enrichment Analysis. GO: Gene Ontology; KEGG: Kyoto Encyclopedia of Genes and Genomes; BP: biological process; CC: molecular function; MF: molecular functions. Supplementary Figure 3. KEGG pathway annotations of the cytokine-cytokine receptor interaction. GO and KEGG were performed by Gene Set Enrichment Analysis. Supplementary Figure 4. The enrichment analysis of TYK2 in STAD (LinkedOmics). (A) A Pearson test was used to analyze correlations between TYK2 and genes differentially expressed in STAD. (B, C) Heat maps showing genes positively and negatively correlated with TYK2 in STAD (Top 50). Red indicates positively correlated genes, and green indicates negatively correlated genes. (D–F) Heat map of GO enrichment in CC terms, BP terms, and MF terms. (G) KEGG pathways analysis. GO and KEGG were performed by Gene Set Enrichment Analysis. GO: Gene Ontology; KEGG: Kyoto Encyclopedia of Genes and Genomes; BP: biological process; CC: molecular function; MF: molecular functions. Supplementary Figure 5. KEGG pathway annotations of the ribosome. KEGG: Kyoto Encyclopedia of Genes and Genomes.
