# Supplementary material for: JAK3 and TYK2 Serve as Prognostic Biomarkers and Are Associated with Immune Infiltration in Stomach Adenocarcinoma
Source: Biomed Res Int. 2020 Sep 19;2020:7973568. doi: 10.1155/2020/7973568 (PMC7559258; doi:10.1155/2020/7973568)
Supplement: Supplementary Materials — Supplementary Figure 1 The expression of JAKs in STAD (UALCAN). The expressions of JAK1, JAK2, JAK3, and TYK2 were significantly elevated in STAD tissues at mRNA level. STAD: stomach adenocarcinoma; ∗∗∗P < 0.001. Supplementary Figure 2. The enrichment analysis of JAK3 in STAD (LinkedOmics). (A) A Pearson test was used to analyze correlations between JAK3 and genes differentially expressed in STAD. (B, C) Heat maps showing genes positively and negatively correlated with JAK3 in STAD (Top 50). Red indicates positively correlated genes, and green indicates negatively correlated genes. (D–F) Heat map of GO enrichment in CC terms, BP terms, and MF terms. (G) KEGG pathways analysis. GO and KEGG were performed by Gene Set Enrichment Analysis. GO: Gene Ontology; KEGG: Kyoto Encyclopedia of Genes and Genomes; BP: biological process; CC: molecular function; MF: molecular functions. Supplementary Figure 3. KEGG pathway annotations of the cytokine-cytokine receptor interaction. GO and KEGG were performed by Gene Set Enrichment Analysis. Supplementary Figure 4. The enrichment analysis of TYK2 in STAD (LinkedOmics). (A) A Pearson test was used to analyze correlations between TYK2 and genes differentially expressed in STAD. (B, C) Heat maps showing genes positively and negatively correlated with TYK2 in STAD (Top 50). Red indicates positively correlated genes, and green indicates negatively correlated genes. (D–F) Heat map of GO enrichment in CC terms, BP terms, and MF terms. (G) KEGG pathways analysis. GO and KEGG were performed by Gene Set Enrichment Analysis. GO: Gene Ontology; KEGG: Kyoto Encyclopedia of Genes and Genomes; BP: biological process; CC: molecular function; MF: molecular functions. Supplementary Figure 5. KEGG pathway annotations of the ribosome. KEGG: Kyoto Encyclopedia of Genes and Genomes. [file 7973568.f1.zip › 7973568.f1/Supplementary Fig 1.pdf]

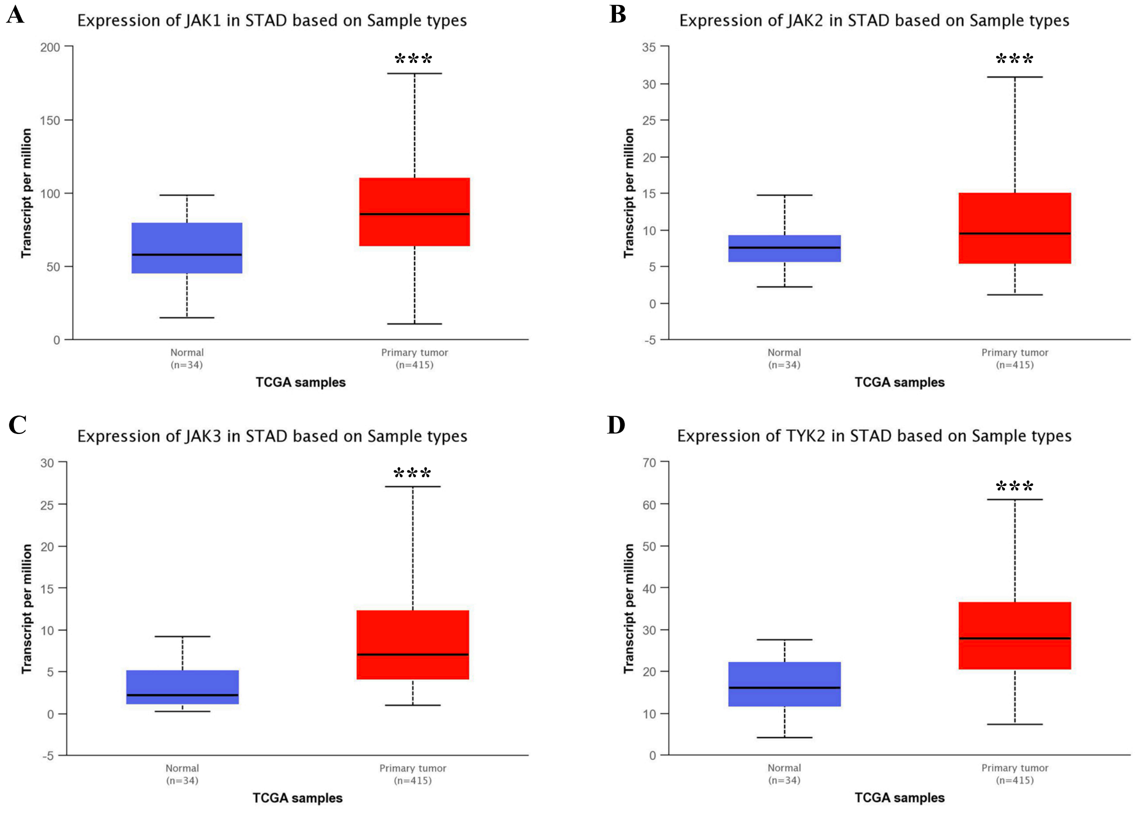
A
Expression of JAK1 in STAD based on Sample types
Transcript per million
200
150
100
50
0
***
Normal
(n=34)
Primary tumor
(n=415)
TCGA samples
B
Expression of JAK2 in STAD based on Sample types
Transcript per million
35
30
25
20
15
10
5
0
-5
***
Normal
(n=34)
Primary tumor
(n=415)
TCGA samples
C
Expression of JAK3 in STAD based on Sample types
Transcript per million
30
25
20
15
10
5
0
-5
***
Normal
(n=34)
Primary tumor
(n=415)
TCGA samples
D
Expression of TYK2 in STAD based on Sample types
Transcript per million
70
60
50
40
30
20
10
0
***
Normal
(n=34)
Primary tumor
(n=415)
TCGA samples
