# Supplementary figures and images for: JAK3 and TYK2 Serve as Prognostic Biomarkers and Are Associated with Immune Infiltration in Stomach Adenocarcinoma
Source: Biomed Res Int. 2020 Sep 19;2020:7973568. doi: 10.1155/2020/7973568 (PMC7559258; doi:10.1155/2020/7973568)

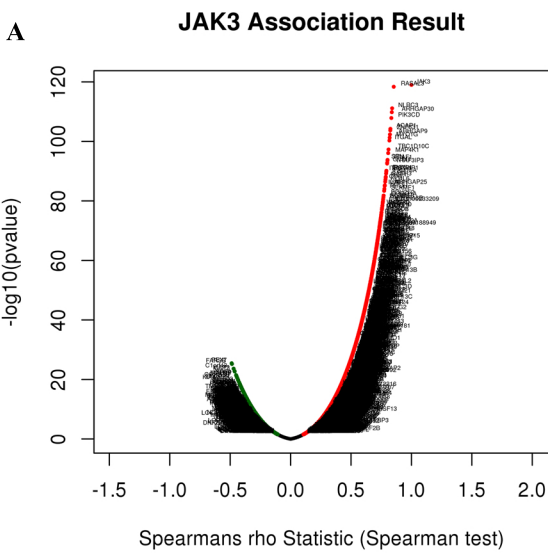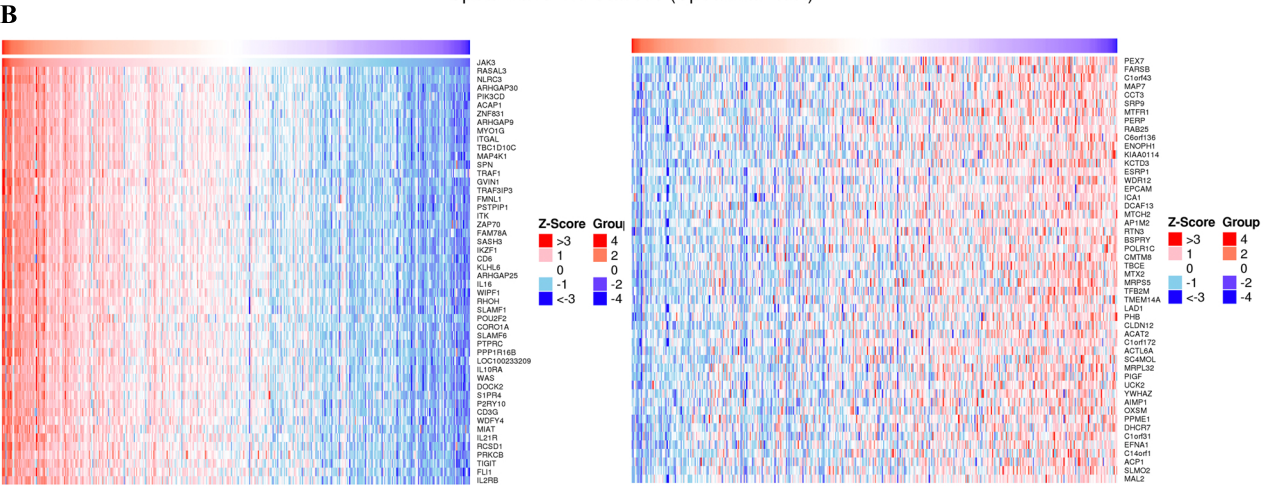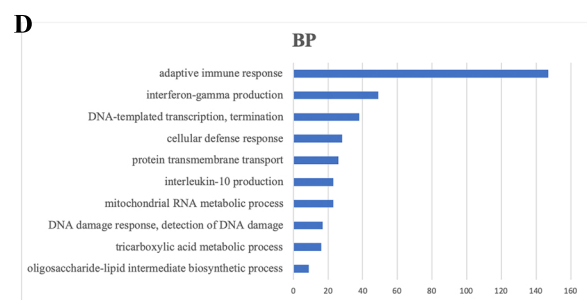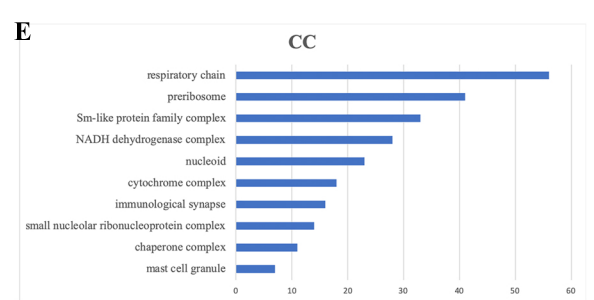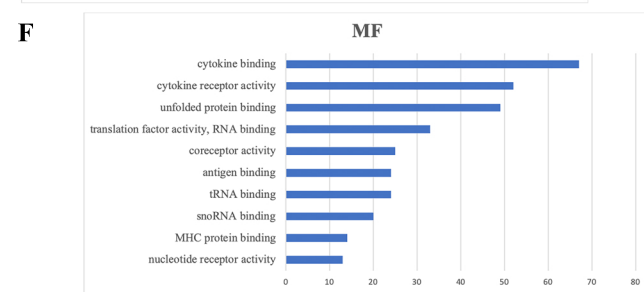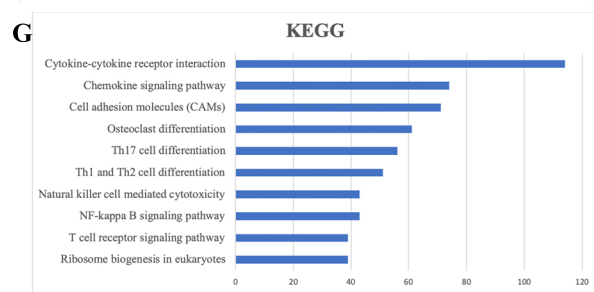

Supplement: Supplementary Materials — Supplementary Figure 1 The expression of JAKs in STAD (UALCAN). The expressions of JAK1, JAK2, JAK3, and TYK2 were significantly elevated in STAD tissues at mRNA level. STAD: stomach adenocarcinoma; ∗∗∗P < 0.001. Supplementary Figure 2. The enrichment analysis of JAK3 in STAD (LinkedOmics). (A) A Pearson test was used to analyze correlations between JAK3 and genes differentially expressed in STAD. (B, C) Heat maps showing genes positively and negatively correlated with JAK3 in STAD (Top 50). Red indicates positively correlated genes, and green indicates negatively correlated genes. (D–F) Heat map of GO enrichment in CC terms, BP terms, and MF terms. (G) KEGG pathways analysis. GO and KEGG were performed by Gene Set Enrichment Analysis. GO: Gene Ontology; KEGG: Kyoto Encyclopedia of Genes and Genomes; BP: biological process; CC: molecular function; MF: molecular functions. Supplementary Figure 3. KEGG pathway annotations of the cytokine-cytokine receptor interaction. GO and KEGG were performed by Gene Set Enrichment Analysis. Supplementary Figure 4. The enrichment analysis of TYK2 in STAD (LinkedOmics). (A) A Pearson test was used to analyze correlations between TYK2 and genes differentially expressed in STAD. (B, C) Heat maps showing genes positively and negatively correlated with TYK2 in STAD (Top 50). Red indicates positively correlated genes, and green indicates negatively correlated genes. (D–F) Heat map of GO enrichment in CC terms, BP terms, and MF terms. (G) KEGG pathways analysis. GO and KEGG were performed by Gene Set Enrichment Analysis. GO: Gene Ontology; KEGG: Kyoto Encyclopedia of Genes and Genomes; BP: biological process; CC: molecular function; MF: molecular functions. Supplementary Figure 5. KEGG pathway annotations of the ribosome. KEGG: Kyoto Encyclopedia of Genes and Genomes. [file 7973568.f1.zip › 7973568.f1/Supplementary Fig 2.pdf]

## CYTOKINE-CYTOKINE RECEPTOR INTERACTION

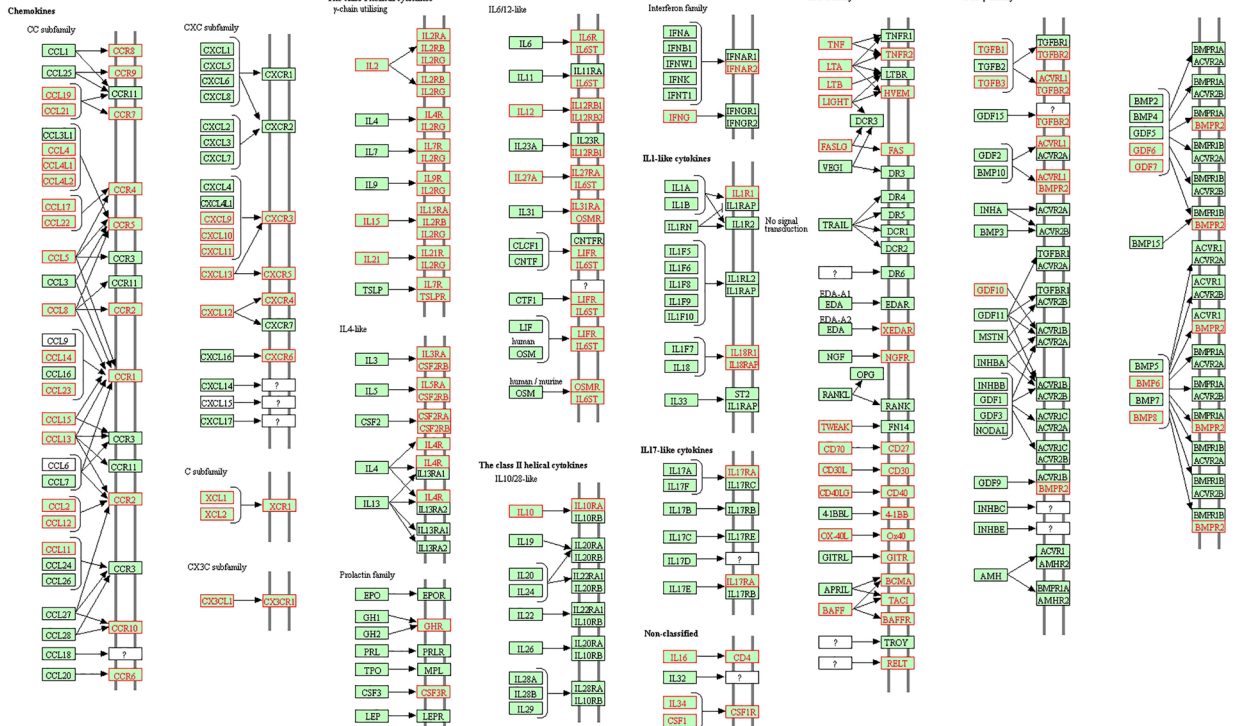

Supplement: Supplementary Materials — Supplementary Figure 1 The expression of JAKs in STAD (UALCAN). The expressions of JAK1, JAK2, JAK3, and TYK2 were significantly elevated in STAD tissues at mRNA level. STAD: stomach adenocarcinoma; ∗∗∗P < 0.001. Supplementary Figure 2. The enrichment analysis of JAK3 in STAD (LinkedOmics). (A) A Pearson test was used to analyze correlations between JAK3 and genes differentially expressed in STAD. (B, C) Heat maps showing genes positively and negatively correlated with JAK3 in STAD (Top 50). Red indicates positively correlated genes, and green indicates negatively correlated genes. (D–F) Heat map of GO enrichment in CC terms, BP terms, and MF terms. (G) KEGG pathways analysis. GO and KEGG were performed by Gene Set Enrichment Analysis. GO: Gene Ontology; KEGG: Kyoto Encyclopedia of Genes and Genomes; BP: biological process; CC: molecular function; MF: molecular functions. Supplementary Figure 3. KEGG pathway annotations of the cytokine-cytokine receptor interaction. GO and KEGG were performed by Gene Set Enrichment Analysis. Supplementary Figure 4. The enrichment analysis of TYK2 in STAD (LinkedOmics). (A) A Pearson test was used to analyze correlations between TYK2 and genes differentially expressed in STAD. (B, C) Heat maps showing genes positively and negatively correlated with TYK2 in STAD (Top 50). Red indicates positively correlated genes, and green indicates negatively correlated genes. (D–F) Heat map of GO enrichment in CC terms, BP terms, and MF terms. (G) KEGG pathways analysis. GO and KEGG were performed by Gene Set Enrichment Analysis. GO: Gene Ontology; KEGG: Kyoto Encyclopedia of Genes and Genomes; BP: biological process; CC: molecular function; MF: molecular functions. Supplementary Figure 5. KEGG pathway annotations of the ribosome. KEGG: Kyoto Encyclopedia of Genes and Genomes. [file 7973568.f1.zip › 7973568.f1/Supplementary Fig 3.pdf]

## A TYK2 Association Result

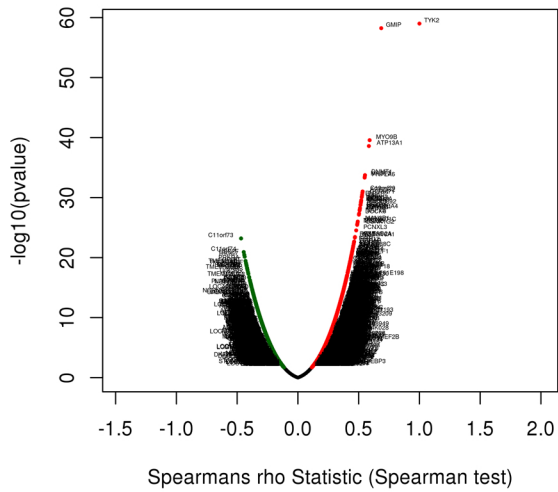

B

C

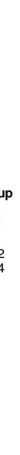

D

E

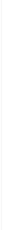

F

G

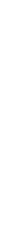

H

I



J

K

Supplement: Supplementary Materials — Supplementary Figure 1 The expression of JAKs in STAD (UALCAN). The expressions of JAK1, JAK2, JAK3, and TYK2 were significantly elevated in STAD tissues at mRNA level. STAD: stomach adenocarcinoma; ∗∗∗P < 0.001. Supplementary Figure 2. The enrichment analysis of JAK3 in STAD (LinkedOmics). (A) A Pearson test was used to analyze correlations between JAK3 and genes differentially expressed in STAD. (B, C) Heat maps showing genes positively and negatively correlated with JAK3 in STAD (Top 50). Red indicates positively correlated genes, and green indicates negatively correlated genes. (D–F) Heat map of GO enrichment in CC terms, BP terms, and MF terms. (G) KEGG pathways analysis. GO and KEGG were performed by Gene Set Enrichment Analysis. GO: Gene Ontology; KEGG: Kyoto Encyclopedia of Genes and Genomes; BP: biological process; CC: molecular function; MF: molecular functions. Supplementary Figure 3. KEGG pathway annotations of the cytokine-cytokine receptor interaction. GO and KEGG were performed by Gene Set Enrichment Analysis. Supplementary Figure 4. The enrichment analysis of TYK2 in STAD (LinkedOmics). (A) A Pearson test was used to analyze correlations between TYK2 and genes differentially expressed in STAD. (B, C) Heat maps showing genes positively and negatively correlated with TYK2 in STAD (Top 50). Red indicates positively correlated genes, and green indicates negatively correlated genes. (D–F) Heat map of GO enrichment in CC terms, BP terms, and MF terms. (G) KEGG pathways analysis. GO and KEGG were performed by Gene Set Enrichment Analysis. GO: Gene Ontology; KEGG: Kyoto Encyclopedia of Genes and Genomes; BP: biological process; CC: molecular function; MF: molecular functions. Supplementary Figure 5. KEGG pathway annotations of the ribosome. KEGG: Kyoto Encyclopedia of Genes and Genomes. [file 7973568.f1.zip › 7973568.f1/Supplementary Fig 4.pdf]
